# Relationship Status and Quality Associations with HIV Care Cascade Outcomes Among Sexual Minority Men Living with HIV in the US: Indications of a Dyadic Coping Paradox

**DOI:** 10.1007/s10461-026-05050-4

**Published:** 2026-03-11

**Authors:** Joseph R. Hillesheim, Tyrel J. Starks

**Affiliations:** 1 Department of Psychology, Hunter College of the City University of New York, 695 Park Ave. 611 Hunter North, New York, NY 10065 USA; 2 Doctoral Program in Health Psychology and Clinical Science, Graduate Center of the City University of New York, New York, NY USA; 3 Department of Psychology, Stony Brook University, Stony Brook, New York 11794 USA

**Keywords:** Couples, AIDS, Anti-retroviral treatment, Drug use, Cannabis, Sexually transmitted infections

## Abstract

Dyadic coping among sexual minority men (SMM) has been a focus of HIV prevention research for decades, but few studies have examined relationship quality as a covariate of HIV care cascade outcomes (ART adherence and an undetectable viral load (VL)). This study utilized a 5-category relationship status variable (single; non-monogamous, sero-discordant; non-monogamous, sero-concordant; monogamous, sero-discordant; monogamous, sero-concordant) to test the hypothesis that relationship quality would moderate associations between relationship status and HIV care outcomes. Adult SMM living with HIV (LWHIV) (*n =* 1389), recruited via social networking applications between January and December 2021, completed a cross-sectional, online survey. At average levels of relationship quality, only non-monogamous SMM with sero-discordant partners were more likely to be adherent to ART (*OR* = 3.064, *p*<.001) and have an undetectable VL (*OR* = 2.595, *p*<.001) compared to single SMM. Among non-monogamous SMM with sero-discordant partners, relationship quality was positively associated with ART adherence (*OR* = 1.065, *p*<.001) and having an undetectable VL (*OR* = 1.046, *p*=.003). Among monogamous SMM with sero-concordant partners, the effect of relationship quality on ART adherence (*OR*=0.855, *p*=.007) and having an undetectable VL (*OR*=0.909, *p*=.011) was significantly smaller compared to non-monogamous SMM with sero-discordant partners. Among non-monogamous SMM with sero-concordant partners, the effect of relationship quality on ART adherence (*OR*=0.956, *p*=.039) was also significantly smaller compared to non-monogamous SMM with sero-discordant partners. Results suggest non-monogamous SMM with sero-discordant partners in high-quality relationships may experience the greatest motivation to engage in HIV care. Messages enhancing motivation for care engagement to improve individual health for SMM LWHIV may augment treatment as prevention.

## Introduction

Sexual minority men (i.e., gay, bisexual, and other cisgender men who have sex with men; SMM) accounted for approximately 71% of new HIV infections and 65% of those living with HIV (LWHIV) in the United States (US) in 2022 [1]. Given that they constitute the majority of those LWHIV, it is of particular concern that approximately 40% of SMM LWHIV report suboptimal adherence to antiretroviral therapy (ART) (defined as less than 80–85% adherence to treatment) [2]. As articulated in the HIV care cascade [3], ART adherence, and subsequently achieving VL suppression has the potential to prevent onward HIV transmission and promotes the health and longevity of those LWHIV. People LWHIV who maintain an undetectable VL cannot transmit HIV to sexual partners [4], and life expectancy among those who remain adherent to ART is similar to the general population, particularly among those who began newer treatments during the past 10 years [5].

Research in the first decades of the 21st century called attention to the potential for HIV transmission through sex between main or primary relationship partners [6–8]. It also illustrated the extent to which relationship factors, such as sexual agreements – the rules couples develop governing sex with partners outside of their relationship [9] – regulate sexual behavior and HIV prevention decision making [6–8]. Since that time, factors associated with sexual HIV transmission risk among partnered SMM has been a primary focus of HIV prevention research (see [10] for review) and intervention development (e.g., [11], [12], [13]).

In contrast to the attention garnered by main partner relationships and HIV prevention, relatively fewer studies have examined how relationship partners and relationship factors associated with HIV risk are subsequently associated with HIV care cascade outcomes (e.g., ART adherence and an undetectable VL) among those LWHIV. Existing research suggests that, compared to single SMM, those LWHIV who are in a relationship are more likely to have a regular healthcare provider, have received recent HIV care, and have ever taken an ART [14]. Additionally, qualitative studies of male couples suggest that partner support enhances motivation for medication adherence and VL suppression (e.g., [15], [16]).

More recently, Starks and colleagues [17] found that whether partnered SMM LWHIV were more or less likely than single SMM to achieve key steps in the HIV care cascade – having an ART prescription, ART adherence, and having an undetectable VL – varied with their sexual agreement as well as their partners’ serostatus. Compared to single SMM LWHIV, partnered SMM with non-monogamous agreements (where sex with outside partners is in some way permitted) with sero-discordant (HIV negative) primary partners were significantly more likely to have an ART prescription and to report an undetectable VL. Starks and colleagues [17] speculated that these partnered SMM experience maximum interpersonal motivation to achieve an undetectable VL in order to minimize the risk of onward HIV transmission to either their primary partner or a sex partner outside their relationship. Conversely, partnered SMM with sero-concordant partners were significantly *less* likely to report ART adherence compared to single SMM LWHIV regardless of whether they had a monogamous sexual agreement (restricting sex to their primary relationship partner) or a non-monogamous one [17]. This pattern aligned with a previous finding indicating that SMM LWHIV who have sero-discordant partners have better ART adherence compared to those with concordant partners [14].

The possibility that specific subgroups of partnered SMM LWHIV are disproportionately likely to be lost to the HIV care cascade would be of substantive concern. It would suggest that tailored messaging and intervention strategies may be necessary to address the needs of this group and ensure linkage to and continuity of HIV care. At present, studies in this area are formative and one notable limitation of existing research is that no study has had access to data that included a measure of relationship quality. It is at least plausible that apparent between-group differences observed to date are the result of a failure to control for differences in relationship quality.

Interdependence theory [18, 19] and related theories of dyadic coping and health [20] provide a framework for understanding why relationship quality might be a central predictor of HIV care cascade outcomes for partnered SMM LWHIV. Lewis and colleagues [20] suggest that relationship quality—satisfaction, commitment, and investment—is associated with health enhancing behaviors. High quality relationships contribute to the development of cognitive interdependence also known as relational orientation [21] and the related phenomenon of transformation of motivation. Cognitive interdependence refers to a condition wherein partners in the relationship begin to think in terms of “we” and “us” as membership in the relationship becomes integrated into a personal sense of self [21]; meanwhile, transformation of motivation generally refers to a realignment in partners’ priorities – from a focus on maximizing personal gains to a consideration of one another’s needs and relational outcomes [22]. Applied specifically to health behavior, Lewis and colleagues [20] suggested that the transformation of motivation results in partners reinterpreting individual health threats as shared stressors. Subsequently, transformation of motivation contributes to the initiation and maintenance of health behaviors, such as managing chronic health conditions.

A small number of studies – mostly focused on SMM in sero-discordant relationships LWHIV – have tested assumptions of interdependence theory applied to HIV care cascade outcomes. Aligned with the general premise of Lewis and colleagues [20], greater relational orientation [23] and higher relationship commitment [24] have been associated with greater odds of VL suppression. Similarly, better dyadic communication generally [24] and better communication about sexual agreements specifically [25] have been associated with better ART adherence. Although promising, these findings are limited by a predominant focus on partnered SMM LWHIV in sero-discordant relationships.

Any study of HIV care cascade outcomes among SMM should necessarily account for a number of established correlates. Racial and ethnic disparities have been documented. Black and Latino SMM respectively accounted for 32% and 28% of those LWHIV in the US [1]. SMM of color are less likely to be adherent to ART and achieve VL suppression compared to White SMM [26]. Stimulant drug use, such as methamphetamine, among SMM has been associated with lower odds of engagement in HIV care [27], ART adherence [28], and VL suppression [29, 30]. Cannabis use has a less consistent association with HIV care outcomes. Although some studies have found that cannabis use among people LWHIV broadly has been associated with lower odds of ART adherence [31], other studies have found no association between cannabis use and ART adherence and VL suppression [32].

The purpose of this study was to examine relationship status differences in two HIV care cascade outcomes (ART adherence and having an undetectable VL) in a sample of SMM LWHIV. Relationship status was operationalized as a 5-category nominal variable. Single individuals were distinguished from 4 categories of partnered participants based upon the serostatus of their partners and their sexual agreements: monogamous sero-concordant, monogamous sero-discordant, non-monogamous sero-concordant, non-monogamous sero-discordant. We hypothesized that SMM with a sero-discordant partner and a non-monogamous agreement would be more likely to report ART adherence and an undetectable VL. Additionally, we explored the possibility that relationship quality would moderate the association between relationship status groups and HIV care outcomes (ART adherence and having an undetectable VL). Aligned with interdependence theory, we anticipated that relationship quality would be positively associated with ART adherence and having an undetectable VL for all groups, but that this effect would be greater in magnitude for those partnered SMM with sero-discordant partners and non-monogamous sexual agreements, wherein interpersonal motivation to reduce onward HIV transmission risk is maximized.

## Methods

### Participants

Participants viewed consent information and responded to a survey in English. Eligible participants were aged 18 years or older, identified as cisgender male and reported LWHIV as well as a residence in the US. Among those who indicated having a main partner, participants were included if their main partner identified as cisgender male and was aged 18 or older. Participants were excluded if they were not currently prescribed ART.

### Procedures

Participants responded to advertisements placed on dating/hook-up applications for gay, bi, trans, and queer people. The advertisement included a Qualtrics survey link that directed respondents to consent information, which was displayed in English. Those consenting indicated so by clicking a button to proceed into a brief survey (approximately 11 min) that assessed eligibility for multiple studies funded by the National Institutes of Health. Respondents did not receive compensation for survey completion. The City University of New York Integrated Institutional Review Board reviewed and approved all procedures.

### Measures

#### Demographics

Participants reported their racial identity and ethnic background (Hispanic or Latino vs. not). Those who identified as Hispanic or Latino were categorized as Latino regardless of race. Participants also indicated their age, sexual identity, and zip code which was used to determine geographic region of residence. If partnered, participants reported their main partner’s age and reported their partner’s HIV status using 3 response options: negative, positive, or unknown/unsure. Responses were aggregated into a two-category variable indicating partners’ HIV status (positive or negative/unknown). Finally, participants reported the length of their relationship on an ordinal scale, and it was subsequently dichotomized for analyses (2 years or less versus more than 2 years).

#### Relationship Status and Sexual Agreement

Participants answered two, dichotomous (yes/no) questions assessing relationship status. First, participants were asked, “Are you currently in a relationship?” Those who said “yes” were asked, “Do you consider this partner to be a main partner? By main partner, we mean someone you feel committed to above anyone else. This would be someone you call a boyfriend, partner, significant other, or spouse.” Those who said “yes” to both items were categorized as partnered. Those who said “no” to either question were categorized as single.

Participants answered a single item commonly used in studies of SMM to assess sexual agreements [9], “Which of the following best characterizes how you currently handle sex outside of your relationship?” Participants were categorized as monogamous if they said “neither of us has sex with others, we are monogamous,” or “I don’t have sex with others and I don’t know what my partner does.” Participants were categorized as non-monogamous if they answered “only I have sex with others,” “only he has sex with others,” “both of us have sex with others separately,” “we both have sex with others separately and together,” “I have sex with others and I don’t know what my partner does,” or “both of us have sex with others together.”

Additionally, relationship status, sexual agreement, and partner HIV status variables were integrated to produce a single, 5-category variable: single; partnered, monogamous with a discordant partner; partnered, monogamous with a concordant partner; partnered, non-monogamous with a discordant partner; partnered, non-monogamous with a concordant partner.

#### Relationship Quality

Participants completed three subscales of the Perceived Relationship Quality Component (PRQC) scale [33] to assess relationship quality. Subscales assessed satisfaction (“How happy are you with your relationship?”), commitment (“How dedicated are you to your relationship?”), and intimacy (“How connected are you to your partner?”). Each subscale included 3 items. All 9 items were summed to produce the total relationship quality score. Higher scores indicated better relationship quality. Reliability was excellent (Cronbach’s *α* = 0.946).

#### Drug Use

Participants answered a single item to assess drug use: “Have you used any of the following drugs in the past 30 days?” Response options included cannabis and six categories of illicit drugs (i.e., cocaine or crack, ecstasy or MDMA, gamma hydroxybuterate, ketamine, amphetamines, or psychedelics – LSD, PCP, mescaline or mushrooms). Participants selected all that applied. Responses were aggregated into 3 dichotomous variables indicating whether the participant used cannabis, stimulant drugs (cocaine, crack, or amphetamines), or other illicit drugs (excluding cannabis or stimulants) during the 30-day assessment period.

#### ART Adherence

Participants answered two questions to assess ART adherence. First, participants were asked, “Are you currently prescribed HIV medication?” Those who answered “yes” were asked, “How much of your prescribed HIV medication have you taken in the past month (30 days)?” Participants responded using a visual, sliding scale (0%-100%) which indicated that 0% meant “you have taken no medication,” 50% meant “you have taken half of your medication,” and 100% meant “you have taken every single dose of your medication.” Responses were dichotomized to distinguish between those who took their ART at least 80% of the time or more and those who take their ART less than 80% of the time.

#### Undetectable VL

An undetectable VL was defined as having a most-recent VL test result obtained in the past 6-months that was undetectable. Participants therefore answered two items assessing VL. First, participants were asked, “When was your most recent viral load test?” Response options included: “Within the past month,” “1–3 months ago,” “4–6 months ago,” “7–12 months ago,” and “over a year ago.” Participants were also asked, “What were the results of your most recent viral load test?” Response options included: “Undetectable, “detectable,” and “not sure/don’t remember.” Responses were combined to create a dichotomous variable indicating whether participants reported an undetectable VL that was obtained in the past 6-months (versus those who reported a detectible or uncertain VL test result and those who had not had a VL test in the past 6-months).

### Analytic Plan

All analyses were conducted with SPSS (Version 29). Data were reviewed for duplicates based on IP address, contact information, and demographic responses. Survey responses were also monitored by Qualtrics fraud detection. Only initial records were retained when duplicates were identified.

Two logistic regression analyses predicted ART adherence and VL detectability. Each analysis comprised a sequence of models testing the significance of interaction terms. First, an initial model was calculated containing only main effects. An omnibus log-likelihood *χ*^*2*^ difference test evaluated the significance of this model relative to a null model (in which all regression coefficients were constrained to be zero). A second model incorporated interaction terms between composite relationship status variables (monogamous with a concordant partner, monogamous with a discordant partner, non-monogamous with a concordant partner, or non-monogamous with a discordant partner) and relationship quality. Log-likelihood *χ*^*2*^ difference tests determined whether the second model provided significantly better fit compared to the initial model including only main effects. A final omnibus log-likelihood *χ*^*2*^ test evaluated the significance of the final model compared to a null model (wherein all regression coefficients were constrained to be zero).

Single respondents were systematically missing data for relationship-related variables (relationship quality and length as well as main partner descriptive information). These were incorporated into multivariable models using established procedures for hurdle covariates [34, 35]. First, continuous variables (relationship quality and main partner age) were mean centered. Then, respondents who reported being single (and were missing these data) were assigned values of zero. Similarly, categorical variables (relationship length and main partner race and ethnicity) were referent dummy coded and single people were assigned values of zero. Once prepared, these hurdle covariates were incorporated into multivariable models accompanied by dummy codes indicating relationship status as well as sexual agreement and partner serostatus group.

Typical hurdle covariate procedures ensure that regression coefficients for relationship covariates quantify the association between that respective predictor and the outcome among partnered respondents. The hypothesis that sexual agreement and partner serostatus would moderate the association between relationship quality and HIV care cascade outcomes was evaluated through the inclusion of 3 interaction terms.

In initial models, the simple main effect of relationship quality was calculated among partnered SMM with sero-discordant partners and non-monogamous sexual agreements. These models included interactions between relationship quality and the other three sexual agreement and partner serostatus groups (monogamous with a concordant partner, monogamous with a discordant partner, non-monogamous with a concordant partner). Where relevant, the simple main effect of relationship quality was subsequently evaluated for other partner serostatus and sexual agreement groups by rotating the referent category and changing corresponding interaction terms. Likewise, between relationship status, partner sero-status, and sexual agreement group differences were evaluated in initial models at average levels of relationship quality. Where relevant, these between group differences were subsequently evaluated at high (1 SD above the mean) and low (1 SD below the mean) levels of relationship quality by centering the variable at the desired level (while single people were missing) and subsequently assigning single people a value of zero before utilizing the prepared variable as a hurdle covariate.

**Fig. 1:**
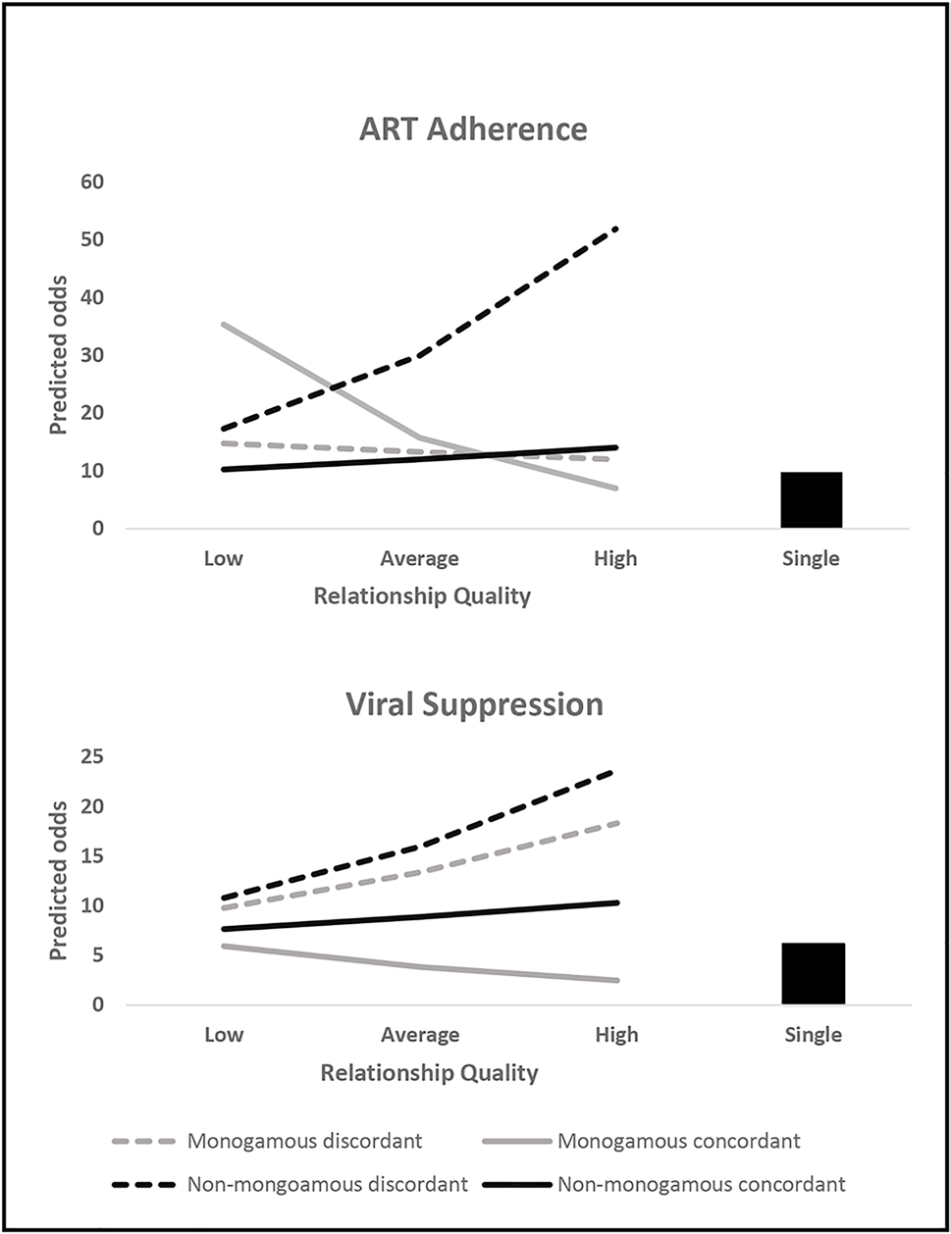
Predicted odds of ART adherence and viral suppression

## Results

Between January and September 2021 recruitment advertisements yielded 22,263 survey starts and 10,258 (46.1%) complete records. After quality assurance review, 762 (7.4%) records were removed, including 526 (5.1%) flagged by Qualtrics as fraudulent and 236 (2.3%) identified during manual review as likely duplicates. This process produced 9,496 valid records, and 1,389 of these met criteria for study inclusion. Records were excluded if respondents were under age 18 (*n* = 203), did not live in the US (*n* = 145), did not identify as cisgender male (*n* = 812), indicated that their main partner was under age 18 or did not identify as cisgender male (*n* = 631), or indicated that their HIV status was negative or unknown (*n* = 6,275). Finally, 41 otherwise eligible participants were excluded because they did not have a current ART prescription.

Table 1 displays descriptive information about the analytic sample (*n* = 1389). The average age of respondents was 42.53 years (*SD* = 11.52). The average age of respondents’ partners was 40.65 (*SD* = 11.78). Overall, 20% of respondents identified as Hispanic or Latino. Among those participants who did not identify as Hispanic or Latino, most (47.4%) identified as White; 25% identified as Black; 5.9% identified as multiracial or identified in some other way; and 1.7% identified as Asian or Pacific Islander. Most participants identified as gay (87.5%); 9.4% identified as bisexual; and 3.1% identified their sexuality in some other way. A plurality (37.1%) lived in the Southeast US; 25.3% lived in the Northeast; 21.7% lived in the West; and 15.9% lived in the Midwest.

**Table 1:** Demographic characteristics

Relationship status	Overall	Single	Partnered monogamous		Partnered non-monogamous				
Partner sero-status			Discordant	Concordant	Discordant	Concordant	χ2	*p*	
n(%)	1389 (100.0)	454 (32.7)	78 (5.6)	59 (4.2)	434 (31.2)	364 (26.2)			
Race							127.35 (16)	< 0.001	
White	658 (47.4)	167 (36.8)^a^	29 (37.2)^a^	17 (28.8)^a^	264 (60.8)^b^	181 (49.7)^c^			
Black	347 (25)	167 (36.8)^a^	25 (32.1)^a, b^	31 (52.5)^c^	44 (10.1)^d^	80 (22)^b^			
Latino	278 (20)	82 (18.1)^a^	14 (17.9)^a^	9 (15.3)^a^	94 (21.7)^a^	79 (21.7)^a^			
Asian	24 (1.7)	9 (2)^a^	3 (3.8)^a^	1 (1.7)^a^	7 (1.6)^a^	4 (1.1)^a^			
Multiracial and Other	82 (5.9)	29 (6.4)^a^	7 (9)^a^	1 (1.7)^a^	25 (5.8)^a^	20 (5.5)^a^			
Sexual Identity							3.76 (8)	0.878	
Gay	1215 (87.5)	388 (85.5)^a^	68 (87.2)^a^	51 (86.4)^a^	389 (89.6)^a^	319 (87.6)^a^			
Bisexual	131 (9.4)	49 (10.8)^a^	8 (10.3)^a^	6 (10.2)^a^	34 (7.8)^a^	34 (9.3)^a^			
Other	43 (3.1)	17 (3.7)^a^	2 (2.6)^a^	2 (3.4)^a^	11 (2.5)^a^	11 (3)^a^			
Geographic Region							28.78 (12)	0.004	
Northeast	351 (25.3)	104 (22.9)^a^	17 (21.8)^a, b^	13 (22)^a, b^	127 (29.3)^b^	90 (24.7)^a, b^			
Midwest	221 (15.9)	78 (17.2)^a^	11 (14.1)^a, b^	14 (23.7)^a^	74 (17.1)^a^	44 (12.1)^b^			
Southeast	516 (37.1)	192 (42.3)^a^	32 (41)^a, b^	24 (40.7)^a, b^	136 (31.3)^b^	132 (36.3)^a, b^			
West	301 (21.7)	80 (17.6)^a^	18 (23.1)^a, b^	8 (13.6)^a^	97 (22.4)^a, b^	98 (26.9)^b^			
	*M(SD)*	*M(SD)*	*M(SD)*	*M(SD)*	*M(SD)*	*M(SD)*			
Age	42.53 (11.52)	42.03 (11.53)	40.68 (10.92)	39.85 (11.76)	43.62 (11.94)	42.69 (10.97)	F(4, 1384) = 2.52	0.039	
Partner age	40.65 (11.78)	-	36 (11.09)^a^	38.49 (11.08)^a, b^	40.87 (11.98)^b^	41.74 (11.54)^b^	F(3, 931) = 5.90	< 0.001	

When omnibus χ2 tests were significant, pairwise comparisons were conducted using the SPSS custom tables function. When omnibus *F* tests were significant, pairwise comparisons were conducted using Bonferroni post hoc tests. Within rows, subgroups with different superscripts differed significantly at *p* < .05.

Table 2 displays descriptive data for primary outcomes as well as substance use. Most participants (84.6%) reported that they took at least 80% of their ART medication in the past 30 days, and 83.8% reported that their last VL result was undetectable and obtained in the past 6 months. About half the sample (50.8%) reported cannabis use in the previous 30 days; about a third (33.5%) reported the use of stimulant drugs; and 21.7% reported the use of at least one other illicit drug.

**Table 2:** Demographic characteristics

Relationship status	Overall	Single	Partnered monogamous		Partnered non-monogamous			
Partner sero-status			Discordant	Concordant	Discordant	Concordant	*χ* ^*2*^	*p*
n(%)	1389 (100.0)	454 (32.7)	78 (5.6)	59 (4.2)	434 (31.2)	364 (26.2)		
ART Adherence								
Less than 80%	214 (15.4)	105 (23.1)^a^	11 (14.1)^a, b^	8 (13.6)^a, b,c^	31 (7.1)^c^	59 (16.2)^b^	43.94 (4)	< 0.001
80% or more	1175 (84.6)	349 (76.9)^a^	67 (85.9)^a, b^	51 (86.4)^a, b,c^	403 (92.9)^c^	305 (83.8)^b^		
Viral Load							33.4 (4)	< 0.001
Detectable or unknown	225 (16.2)	100 (22.0)^a^	9 (11.5)^b, c^	16 (27.1)^a^	40 (9.2)^c^	60 (16.5)^b^		
Undetectable	1164 (83.8)	354 (78.0)^a^	69 (88.5)^b, c^	43 (72.9)^a^	394 (90.8)^c^	304 (83.5)^b^		
Drug use								
Cannabis	706 (50.8)	229 (50.4)^a^	39 (50.0)^a^	30 (50.8)^a^	210 (48.4)^a^	198 (54.4)^a^	2.94 (4)	0.568
Stimulant drugs	465 (33.5)	182 (40.1)^a^	21 (26.9)^b^	8 (13.6)^b^	104 (24.0)^b^	150 (41.2)^a^	48.34 (4)	< 0.001
Other illicit drugs	302 (21.7)	99 (21.8)^a^	4 (5.1)^b^	6 (10.2)^b, c^	84 (19.4)^a, c^	109 (29.9)^d^	33.15 (4)	< 0.001
Relationship length							22.15 (3)	< 0.001
less than 2 years	299 (32.0)	-	40 (51.3)^a^	27 (45.8)^a^	131 (30.2)^b^	101 (27.7)^b^		
2 years or more	636 (68.0)	-	38 (48.7)^a^	32 (54.2)^a^	303 (69.8)^b^	263 (72.3)^b^		
	*M(SD)*		*M(SD)*	*M(SD)*	*M(SD)*	*M(SD)*		
Relationship Quality	51.14 (10.62)	-	53.38 (9.92)	51.69 (10.32)	51.07 (10.49)	50.64 (10.95)	F(3, 931) = 1.49	0.216

*ART*  Antiretroviral therapy. When omnibus χ2 tests were significant, pairwise comparisons were conducted using the SPSS custom tables function. When omnibus *F* tests were significant, pairwise comparisons were conducted using Bonferroni post hoc tests. Within rows, subgroups with different superscripts differed significantly at *p* < .05

### Between Group Differences: Relationship Status, Sexual Agreements and Sero-Concordance

Table 2 provides demographic information for single SMM (*n* = 454, 32.7%) as well as 4 subgroups of partnered SMM, including those with: a monogamous sexual agreement and sero-concordant partner (*n* = 59; 4.2%), a monogamous sexual agreement and sero-discordant partner (*n* = 78; 5.6%), a non-monogamous sexual agreement and sero-concordant partner (*n* = 364, 26.2%), and a non-monogamous sexual agreement and sero-discordant partner (*n* = 434, 31.2%). This subgroup distribution means that most participants were in a non-monogamous relationship (57.4%). Furthermore, 36.8% of respondents in relationships reported that their partners’ HIV status was negative or unknown (sero-discordant).

Significant differences in racial and ethnic composition were observed across relationship status, sexual agreement, and partner serostatus groups – specifically with respect to not Hispanic White and Black participants. SMM in non-monogamous relationships were significantly more likely to identify as White and not Hispanic compared to single SMM and those in monogamous relationships (regardless of partner serostatus). SMM in non-monogamous relationships with sero-discordant partners were then significantly more likely to identify as White and not Hispanic (60.8%) even when compared to SMM in non-monogamous relationships with sero-concordant partners (49.7%). In contrast, monogamous SMM with sero-concordant partners were most likely to identify as Black and not Hispanic (52.5%), followed by those who were single (36.8%), and then those in a non-monogamous relationship with a concordant partner (22.0%). (The odds of identifying as Black and not Hispanic did not differ significant for those in monogamous relationships with sero-discordant partners, 32.1%, compared to those who were single or those in a non-monogamous relationship with a concordant partner.) Partnered SMM in non-monogamous relationships with discordant partners were the least likely to identify as Black and not Hispanic (10.1%). The odds of identifying as Hispanic, Asian and not Hispanic, or in another way (and not Hispanic) did not differ significantly across groups. Although groups did not differ with respect to participants’ personal age, partnered SMM in monogamous relationships with sero-discordant partners had significantly younger partners (*M* = 36, *SD* = 11.09 years) compared to those in non-monogamous relationships with sero-discordant (*M* = 40.87, *SD* = 11.98 years) and sero-concordant (*M =* 41.74, *SD* = 11.54) partners. Those in monogamous relationships with a sero-concordant partner reported average partner ages that did not differ from any other group. Partnered SMM subgroups did not differ significantly with respect to relationship quality. Those with non-monogamous sexual agreements were significantly more likely to be in their relationships for 2 years or more compared to those with monogamous sexual agreements (regardless of partner serostatus).

Partnered SMM with non-monogamous agreements and sero-discordant partners were significantly more likely to be adherent to ART (92.9%) compared to those with non-monogamous agreements and sero-concordant partners (83.8%) as well as those who were single (76.9%). Those partnered SMM with monogamous agreements and sero-concordant as well as discordant partners did not differ significantly from any other group. (Respectively, 86.4% and 85.9% reported 80% ART adherence or better.) Partnered SMM with non-monogamous agreements and sero-discordant partners were also significantly more likely to report an undetectable VL result obtained in the past 6 months (90.8%) compared to those in relationships with non-monogamous agreements and concordant partners (83.5%), monogamous relationships and concordant partners (72.9%), as well as single SMM (78.0%). Partnered SMM with monogamous agreements and sero-discordant partners (88.5% reported an undetectable VL) did not differ significantly from those in non-monogamous relationships with sero-discordant partners or those in non-monogamous relationships with sero-concordant partners. Single SMM and partnered SMM in non-monogamous relationships with sero-concordant partners were significantly more likely to report stimulant use compared to all other groups (40.1% and 41.2% respectively). Partnered SMM in non-monogamous relationships with sero-concordant partners were also significantly more likely to use other illicit drugs compared to all other groups. No between-group differences were statistically significant with respect to cannabis use.

### Multivariable Logistic Regressions: ART Adherence

Table 3 contains the results of the model predicting ART adherence. The initial model, containing only main effects, was superior to a null model (log-likelihood *χ*^*2*^(19) = 172.774, *p*< .001). The final model, including 3 interaction terms, had significantly better model fit compared to the main effects only model (log-likelihood *χ*^*2*^(3) = 12.732, *p*=.005).

Among SMM LWHIV with non-monogamous sexual agreements and discordant partners, relationship quality was positively associated with ART adherence (*OR* = 1.065, *p<*.001). Inspection of interaction terms indicated that the association between relationship quality and ART adherence was significantly smaller among SMM in monogamous relationships with sero-concordant partners (*OR*=0.855, *p*=.007) and among SMM in non-monogamous relationships with sero-concordant partners (*OR*=0.956, *p*=.039). Interaction terms suggested that the association between relationship quality and ART adherence among partnered SMM with monogamous agreements and sero-discordant partners did not differ from that observed for those in relationships with non-monogamous agreements and sero-discordant partners (*OR*=0.928, *p*=.059).

Rotation of referent categories revealed that data only partially conformed to hypotheses related to the anticipated association between relationship quality and ART adherence. The simple main effect of relationship quality was non-significant among partnered SMM in monogamous relationships with sero-concordant partners (*OR*=0.911, *p*=.090); monogamous relationships with sero-discordant partners (*OR*=0.989, *p*=.747), and non-monogamous relationships with concordant partners (*OR* = 1.018, *p*=.180).

At average levels of relationship quality, only those partnered SMM with non-monogamous sexual agreements and sero-discordant partners had significantly better ART adherence compared to those who were single (*OR* = 3.064, *p*<.001). No other subgroups of partnered SMM differed significantly from single SMM. At low levels of relationship quality (1 SD below the mean), none of the partnered SMM subgroups differed significantly from single SMM. Even at high levels of relationship quality, only those partnered SMM with non-monogamous agreements and sero-discordant partners were significantly more likely to be adherent to ART compared to single SMM (*OR* = 6.004, *p*<.001). Differences between single SMM and those in relationships with monogamous agreements and sero-concordant partners (*OR* = 0.600, *p*=.332), monogamous agreements and sero-discordant partners (*OR* = 1.205, *p*=.703), and non-monogamous agreements and sero-concordant partners (*OR* = 1.485, *p*=.172) were uniformly non-significant. (See Fig 1.)

Separately, participants who reported recent stimulant drug use were significantly less likely to report ART adherence (*OR*=0.270, *p*<.001). Cannabis and other illicit drug use were not significantly associated with ART adherence. Two covariates also contributed significantly to the model. Participant age was positively associated with ART adherence, and those who identified as Black were significantly less likely to be adherent to ART compared to those who identified as White.

**Table 3:** Logistic regressions predicting ART adherence and an undetectable VL

	ART adherence			Undetectable VL		
	OR	95% CI	p	OR	95% CI	p
Intercept	1.943	(0.699, 5.403)	0.203	2.039	(0.766, 5.426)	0.154
Individual covariates						
Age	1.039	(1.021, 1.057)	< 0.001	1.026	(1.010, 1.043)	0.002
Partner age	0.990	(0.968, 1.012)	0.360	0.999	(0.979, 1.020)	0.942
Race and ethnicity (ref = White)						
Black	0.636	(0.414, 0.975)	0.038	0.934	(0.617, 1.416)	0.749
Latino	0.671	(0.427, 1.054)	0.083	0.907	(0.585, 1.405)	0.661
Asian	0.667	(0.219, 2.033)	0.477	0.588	(0.206, 1.680)	0.322
Multiracial or other	0.728	(0.373, 1.422)	0.353	0.952	(0.492, 1.842)	0.884
Sexual Orientation (ref = gay)						
Bisexual or identifies another way	0.793	(0.491, 1.282)	0.344	1.253	(0.807, 1.944)	0.315
Geographic Region (ref = Northeast)						
Midwest	1.437	(0.854, 2.418)	0.172	0.548	(0.343, 0.874)	0.012
South	1.191	(0.797, 1.779)	0.393	1.089	(0.722, 1.643)	0.685
West	1.429	(0.888, 2.297)	0.141	0.958	(0.604, 1.517)	0.854
Drug use						
Cannabis	0.861	(0.622, 1.194)	0.370	1.004	(0.735, 1.372)	0.979
Stimulant drug use	0.270	(0.185, 0.395)	< 0.001	0.271	(0.188, 0.392)	< 0.001
Club drug use	0.904	(0.603, 1.353)	0.622	1.254	(0.838, 1.878)	0.272
Relationship covariates						
Relationship length	1.582	(0.992, 2.524)	0.054	0.994	(0.638, 1.549)	0.978
Relationship status						
Monogamous concordant	1.609	(0.568, 4.556)	0.371	0.625	(0.301, 1.297)	0.207
Monogamous discordant	1.361	(0.634, 2.922)	0.429	2.173	(0.957, 4.933)	0.064
Non-monogamous concordant	1.227	(0.759, 1.983)	0.403	1.442	(0.892, 2.331)	0.135
Non-monogamous discordant	3.064	(1.713, 5.480)	< 0.001	2.595	(1.526, 4.411)	< 0.001
Relationship quality	1.065	(1.029, 1.103)	< 0.001	1.046	(1.015, 1.077)	0.003
Interactions						
Monogamous concordant x Relationship Quality	0.855	(0.764, 0.958)	0.007	0.909	(0.845, 0.978)	0.011
Monogamous discordant x Relationship Quality	0.928	(0.858, 1.003)	0.059	0.991	(0.921, 1.066)	0.806
Non-monogamous concordant x Relationship Quality	0.956	(0.915, 0.998)	0.039	0.972	(0.935, 1.010)	0.151

*ART*  Antiretroviral therapy, *VL * Viral load.

### Multivariate Logistic Regressions: Undetectable VL

Table 3 contains the results of the model predicting an undetectable VL. The initial model, containing only main effects, was superior to a null model (log-likelihood *χ*^*2*^(19) = 132.168, *p*< .001). The final model, including 3 interaction terms, had significantly better model fit compared to the main effects only model (log-likelihood *χ*^*2*^(3) = 7.850, *p*=.049).

Among SMM LWHIV with non-monogamous sexual agreements and discordant partners, relationship quality was positively associated with reporting a recent undetectable VL (*OR* = 1.046, *p*=.003). Inspection of interaction terms indicated that the association between relationship quality and an undetectable VL was significantly smaller among SMM in monogamous relationships with sero-concordant partners (*OR*=0.909, *p*=.011); meanwhile, the association between relationship quality and an undetectable VL among partnered SMM with monogamous agreements and sero-discordant partners (*OR*=0.991, *p*=.806) as well as among SMM in relationship with non-monogamous agreements and concordant partners (*OR*=0.972, *p*=.151) did not differ significantly from that observed among SMM in non-monogamous relationships with sero-discordant partners.

Rotation of referent categories revealed that data only partially conformed to hypotheses related to the anticipated association between relationship quality and reported VL detectability. The simple main effect of relationship quality was non-significant among partnered SMM in monogamous relationships with sero-concordant partners (*OR*=0.950, *p*=.137); monogamous relationships with sero-discordant partners (*OR* = 1.036, *p*=.303), and non-monogamous relationships with concordant partners (*OR* = 1.016, *p*=.213).

At average levels of relationship quality, only those partnered SMM with non-monogamous sexual agreements and sero-discordant partners were significantly more likely to report a recent undetectable VL compared to those who were single (*OR* = 2.595, *p*<.001). No other subgroups of partnered SMM differed significantly from single SMM. At low levels of relationship quality (1 SD below the mean), none of the partnered SMM subgroups differed significantly from single SMM. In contrast, at high levels of relationship quality (1 SD above the mean), participants in relationships with sero-discordant partners were significantly more likely to report an undetectable VL regardless of whether they had a monogamous (*OR* = 3.164; *p*=.047) or a non-monogamous (*OR* = 4.166; *p*<.001) sexual agreement. In addition, at high levels of relationship quality, participants in monogamous relationships with sero-concordant partners were significantly less likely to report an undetectable VL compared to single SMM (*OR* = 0.364; *p*=.028). Those in non-monogamous relationships with sero-concordant partners did not differ significantly from single SMM (*OR* = 1.713; *p*=.061). (See Fig 1.)

Separately, recent stimulant drug use was negatively associated with an undetectable VL (*OR*=0.271, *p*<.001). Cannabis and the use of other illicit drugs were not. Two covariates also contributed significantly to the model. Participant age was positively associated with an undetectable VL, and participants who lived in the Midwest were significantly less likely to report an undetectable VL compared to those who lived in the Northeast.

## Discussion

This is the first paper to compare HIV cascade outcomes for single and partnered SMM accounting for relationship quality (and other partner and relational covariates). Although findings generally align with previous observations indicating that partnered SMM with non-monogamous agreements and sero-discordant partners have better ART adherence and VL suppression [17], they go further – suggesting that relationship quality may only be positively associated with HIV care cascade outcomes among this group of partnered SMM. Notably, regardless of the level of relationship quality (average or high), only non-monogamous SMM with sero-discordant partners were more likely to report ART adherence compared to single SMM, and at high levels of relationship quality, only SMM with sero-discordant partners (regardless of sexual agreement) were more likely to report an undetectable VL.

These findings suggest a dyadic coping paradox. Being in a high-quality relationship appears to be the most health-enhancing for non-monogamous SMM with sero-discordant partners, aligning with the general premise that better relationships support better HIV care cascade outcomes. These couples likely experience the greatest interpersonal motivation for HIV prevention. In contrast, SMM with concordant partners do not appear to uniformly experience the same health-enhancing benefits from higher quality relationships, and in the context of VL detectability, monogamous SMM with concordant partners actually appeared to experience diminished health outcomes at high levels of relationship quality. Given the absence of HIV transmission risk within these dyads, SMM LWHIV with concordant partners (regardless of sexual agreement) may be less likely to appraise suboptimal ART adherence and VL detectability as significant health threats within their relationship. They may therefore be less motivated to maintain engagement in the HIV care cascade.

These findings partially align with existing literature suggesting that main partner relationships have the potential to regulate health, and sexual health behavior among SMM (e.g., [10]). They also add to a small number of quantitative studies focused on interdependence in the context of HIV care [24, 36]. These findings replicate and extend recent research indicating that ART adherence and VL detectability vary by relationship status, sexual agreements, and partner sero-status among SMM LWHIV [17]. In particular, Starks and colleagues [17] observed a similar pattern in which non-monogamous SMM with sero-discordant partners report better HIV care related outcomes compared to single SMM LWHIV. The current study contextualizes this finding by including relationship quality as a covariate.

Separately, these findings also corroborate previous research indicating that stimulant use is associated with poorer HIV care cascade outcomes [27–30, 37]. In contrast, neither club drug nor cannabis use were associated with HIV care cascade outcomes. Although the association between stimulant use and ART adherence and VL suppression is well-documented, these findings contribute to a smaller, more equivocal body of research focused on cannabis [31, 32] and club drugs [37] among SMM LWHIV. Future research on substance use and the HIV care cascade might consider the frequency and context of drug use to better understand differences in the effect of stimulant, club drug, and cannabis use on ART adherence and VL suppression.

### Clinical Implications

These findings reinforce the need for continued work to develop and disseminate interventions that address drug use among SMM LWHIV. Across all relationship status groups, stimulant drug use was notably associated with adverse HIV care cascade outcomes (lower odds of ART adherence and having an undetectable VL). Although individually delivered interventions for single SMM have been developed to address stimulant use specifically [38, 39], these studies have not examined the extent to which intervention effects are moderated by relationship status (or partner characteristics).

Second, interventions that address the intersection of drug use, relationship quality and HIV care outcomes may be particularly relevant for sero-discordant couples. Although no current intervention model has been tested, Motivational Interviewing with Couples has been used previously to address multiple behavioral targets (e.g., [40]). Existing interventions that address the intersection of HIV prevention and drug use [41–43] and those that address both HIV prevention as well as the care cascade [44, 45] may serve as a starting point for clinicians engaged in such work.

Finally, these data suggest that at least some couples may benefit from intervention activities that enhance the association between relationship quality and HIV care outcomes by catalyzing the transformation of motivation. Such activities might invite partners to consider or imagine the implications of their personal health for one another’s physical, mental, and emotional well-being as well as the well-being of their relationship. For all couples, but particularly those in which both partners are LWHIV, these conversations may need to explore sources of motivation that are grounded in quality of life for those LWHIV in addition to the prevention of onward transmission.

### Limitations and Conclusions

Several limitations are relevant. Data were generated by a cross-sectional survey and cannot support causal inferences. A convenience sample of participants generated these data after being recruited from social networking applications often (though not exclusively) used for locating sexual partners. The sample may therefore over-represent SMM LWHIV who are relatively sexually active. Given the correspondence between sexual behavior and drug use, this may also mean that the sample over-represents SMM who use drugs. Third, the study relied exclusively on self-report data. The rigor of future studies might be enhanced by the inclusion of objective, biological metrics that could directly assess HIV care cascade outcomes (or drug use) or corroborate self-report data. Finally, although the study had access to a large sample size, subgroups of SMM in relationships with monogamous agreements were relatively small. Future studies with access to larger subsamples of SMM in relationships with monogamous sexual agreements may have more power to detect pairwise between-group differences involving these groups.

This study is the first to include relationship quality as a covariate when comparing HIV care cascade outcomes across single SMM LWHIV and subgroups of those in relationships. Findings indicate that relationship quality is positively associated with ART adherence and an undetectable VL only among non-monogamous SMM with sero-discordant partners, supporting previous research suggesting that this group may be most motivated to engage in HIV care compared to single SMM LWHIV. Importantly, this study suggests that tailored interventions are needed to better retain single and some partnered SMM in the HIV care cascade.

## Data Availability

Data not publicly available. Please contact the corresponding author.

## Code Availability

Descriptive statistics were calculated using SPSS version 29.
